# Molecular characterization of the *Haemonchus contortus* phosphoinositide-dependent protein kinase-1 gene (*Hc-pdk-1*)

**DOI:** 10.1186/s13071-016-1351-6

**Published:** 2016-02-03

**Authors:** Fa-Cai Li, Robin B. Gasser, James B. Lok, Pasi K. Korhonen, Li He, Wen-Da Di, Fang-Yuan Yin, Rui Zhou, Yan-Qin Zhou, Jun-Long Zhao, Min Hu

**Affiliations:** State Key Laboratory of Agricultural Microbiology, Key Laboratory of Development of Veterinary Products, Ministry of Agriculture, College of Veterinary Medicine, Huazhong Agricultural University, Wuhan, 430070 Hubei China; Faculty of Veterinary and Agricultural Sciences, The University of Melbourne, Parkville, VIC 3010 Australia; Department of Pathobiology, School of Veterinary Medicine, University of Pennsylvania, Philadelphia, PA 19104 USA

**Keywords:** *Haemonchus contortus*, Transgenesis, Development, *pdk-1* gene

## Abstract

**Background:**

Phosphoinositide-dependent protein kinase-1 (PDK-1), which functions downstream of phosphoinositide 3-kinase (AGE-1) and activates protein kinases of the AGC family, plays critical roles in regulating biology processes, such as metabolism, growth, development and survival. In the free-living nematode *Caenorhabditis elegans*, PDK-1 is a key component of the insulin-like signalling pathway, regulating the entry into and exit from dauer (arrested development). Although it is proposed that similar molecular mechanisms control the transition from the free-living to the parasitic stages of nematodes, nothing is known about PDK-1 in *Haemonchus contortus*, a socioeconomically important gastric nematode of ruminants.

**Methods:**

Here, we isolated and characterized the *pdk-1* gene (*Hc-pdk-1*) and its inferred product (*Hc*-PDK-1) from *H. contortus*. Using in vitro and in vivo methods, we then studied the transcriptional profiles of *Hc-pdk-1* and anatomical gene expression patterns of *Hc*-PDK-1 in different developmental stages of *C. elegans*.

**Results:**

*In silico* analysis of *Hc*-PDK-1 displayed conserved functional domains, such as protein kinase and pleckstrin homology (PH) domains and two predicted phosphorylation sites (Thr226/Tyr229), which are crucial for the phosphorylation of downstream signalling. The *Hc-pdk-1* gene is transcribed in all of the main developmental stages of *H. contortus*, with its highest transcription in the infective third-stage larvae (iL3) compared with other stages. Transgene constructs, in which respective promoters were fused to the coding sequence for green fluorescent protein (GFP), were used to transform *C. elegans*, and to localize and compare the expression of *Hc-pdk-1* and *Ce-pdk-1*. The expression of GFP under the control of the *Hc-pdk-1* promoter was localized to the intestine, and head and tail neurons, contrasting somewhat the profile for the *C. elegans* ortholog, which is expressed in pharynx, intestine and head and tail neurons.

**Conclusions:**

This is the first characterization of *pdk-1/*PDK-1 from a trichostrongyloid nematode. Taken together, the findings from this study provide a first glimpse of the involvement of *Hc-pdk-1* in the insulin-like signalling pathway in *H. contortus*.

**Electronic supplementary material:**

The online version of this article (doi:10.1186/s13071-016-1351-6) contains supplementary material, which is available to authorized users.

## Background

*Haemonchus contortus* is an important strongylid nematode infecting millions of small ruminants (including sheep and goats) globally. The disease (haemonchosis) caused by this nematode leads to major economic losses associated with morbidity, reduced production and mortality in these livestock species. *H. contortus* feeds on blood in the stomach (abomasum), and causes anaemia, oedema and, in extreme cases, death. Anthelmintic treatment has been an essential part of the control of *H. contortus* and related parasites. However, drug-resistance is now widespread in many countries, due to an excessive and often uncontrolled use of anthelmintics [1–4]. Therefore, it is important to work toward developing new anthelmintics and/or vaccines, built on a sound understanding of key molecules in biological pathways of *H. contortus* and related parasites [5, 6].

The infective, third-stage larva (iL3) of *H. contortus* is a motile, free-living stage that is arrested in its development until it enters the host animal, after which it becomes parasitic. Like iL3 of *H. contortus*, the dauer stage of *Caenorhabditis elegans* is also arrested in its development; this stage ceases to feed under unfavourable conditions, such as overcrowding, limited food availability and high environmental temperature, and has an extended lifespan [7]. Interestingly, when environmental conditions improve, *C. elegans* can exit dauer, and continues to develop and reproduce normally [8].

Evolutionarily, both *C. elegans* and *H. contortus* belong to clade V nematodes [9]. Therefore, it has been postulated that the exit from dauer in the free-living nematode (*C. elegans*) and activation of iL3 in the parasitic nematode (*H. contortus*) are governed by analogous molecular mechanisms [10–13]. Dauer is regulated principally by four signalling pathways, one of which is the insulin-like signaling pathway which involves several proteins, including DAF-2 [14], AGE-1 [15], PDK-1 [16], AKT-1/2 [17] and DAF-16 [18–20]. Signalling via DAF-2, AGE-1 and PDK-1 activates AKT-1/2 by phosphorylation, which, in turn, phosphorylates and, thus, negatively regulates DAF-16, a core regulator of multiple biological processes, such as longevity, stress-resistance and developmental arrest [21, 22].

Although advances have been made in understanding the molecular mechanisms of the dauer state, very little is known about the analogous process of iL3 development in parasitic nematodes. The genomes and transcriptomes of *H. contortus* [23, 24] provide a solid foundation for exploring key molecules in this transition process, but a lack of effective genetic and in vitro culture methods restricts somewhat the study of molecular mechanisms of these nematodes [25–27]. In contrast, *C. elegans* has been used as a surrogate system to explore molecular functions in some parasitic nematodes, such as *H. contortus* [28–30], *Ancylostoma caninum* [31, 32] and *Strongyloides stercoralis* [33, 34]. In the present study, we isolated and characterized the *pdk-1* ortholog from *H. contortus* (*Hc-pdk-1*). Using in vitro and in vivo methods, we studied the transcriptional profiles of *Hc-pdk-1* and anatomical gene expression patterns of *Hc*-PDK-1 in different developmental stages of *C. elegans*.

## Methods

### Ethics statement

All of the experimental animals used in this project were treated in strict accordance with Guidelines for the Use of Experimental Animals in the People’s Republic of China. The production of *H. contortus* in goats was approved by the Animal Ethics Committee of Hubei Province (permit SYXK-0029).

### Nematode strains and their maintenance

The *H. contortus* Haecon 5 strain was maintained by serial passage in goats (helminth-free), which were infected intra-ruminally with 8000 iL3. Eggs, first-stage larvae (L1s), second-stage larvae (L2s) and iL3s were harvested or cultured from the faeces from infected goats, as described previously [35, 36]. L4s and adults were collected from the abomasa from infected goats euthanized at 8 and 30 days, respectively. These two developmental stages were washed extensively in phosphate-buffered saline (PBS; pH 7.4), and male and female worms were separated prior to storage at −80 °C. The N2 strain of *C. elegans* was obtained from the *Caenorhabditis* Genetics Center (CGC, University of Minnesota, USA) and maintained using standard procedures [37].

### DNA and RNA preparation

Genomic DNA samples were extracted from mixed stages of *C. elegans* or L3s of *H. contortus* using the EasyPure Genomics DNA Kit (TransGen Biotech, China). Total RNA samples were isolated separately from egg, L1, L2, iL3, female and male fourth-stage larvae (L4s), and female and male adult stages of *H. contortus* using the TRIzol Plus Purification kit (Life Technologies, USA). RNA yields and quality were verified by spectrophotometric (NanoDrop Technologies) and by electrophoretic analysis, respectively. RNA was treated with RQ1-RNase-Free DNase (Promega, USA). Following isolation, nucleic acid samples were immediately frozen and stored at −80 °C.

### Isolation of the *Hc-pdk-1* gene and its upstream region

Guided by genomic and transcriptomic data for *H. contortus* (see [24]; GenBank accession no. AUUS00000000; SRA; accession nos. SRP027504 and SRP026668), we isolated the full-length *Hc-pdk-1* gene and its cDNA (GenBank accession no. KU522003). The coding region was amplified and sequenced using the primer pair Hc-pdk-F and Hc-pdk-R (Additional file 1), and then cloned into the pMD-19 T vector (Takara, Japan). The gene sequence was obtained from the *H. contortus* genome [24], and exon-intron boundaries established. Part of the 5′-flanking region of the *Hc-pdk-1* coding sequence was amplified from genomic DNA of *H. contortus* using the primers Hc-pdk-gw-1R and Hc-pdk-gw-2R (Additional file 1) using the GenomeWalker Universal Kit (Clontech, USA; according to the protocol provided) and sequenced (GenBank accession no. KU522003). Subsequently, the entire upstream sequence was amplified using the primer set Hc-pdk-pro-F/Hc-pdk-gfp-R (Additional file 1), cloned into the pMD-19T vector and sequenced in both directions (Sangon Biotech, Shanghai).

### Bioinformatic analyses

Nucleotide sequences were assembled using the program CAP3 (http://bio.ifom-ieo-campus.it/) and compared with those in non-redundant databases using the BLAST v.2.0 suite of programs from the National Center for Biotechnology Information (NCBI) (http://www.ncbi.nlm.nih.gov/BLAST), the Sanger Centre (www.sanger.ac.uk) and the Parasite Genome database (www.ebi.ac.uk) to confirm the identity of genes isolated. Individual cDNAs were conceptually translated using the selection “translate”, available at http://bioinformatics.org/. Protein motifs were identified by scanning the databases Pfam (www.sanger.ac.uk/Software/Pfam) and PROSITE (www.expasy.ch/tools/scnpsit1.html). Signal sequences were predicted using SignalP v.2.0 [38], available at the Center for Biological Sequence Analysis (www.cbs.dtu.dk/services/SignalP). Amino acid sequences were aligned using the program Clustal W [39] and adjusted manually. Promoter elements in the 5′-genomic region upstream of the start codon (ATG) of *Hc-pdk-1* were predicted using the transcription element search system (PLACE; available at http://www.dna.affrc.go.jp/PLACE/signalscan.html).

The predicted amino acid sequences of *Hc*-PDK-1 and homologues from other invertebrates (nematodes and *Drosophila melanogaster*) and vertebrates (human and *Xenopus laevis*) were aligned and subjected to phylogenetic analyses. These analyses were conducted using the neighbor-joining (NJ), maximum parsimony (MP) and maximum likelihood (ML) methods, respectively, based on the Jones-Taylor-Thornton (JTT) model [40]. Confidence limits were assessed using a bootstrap procedure employing 1000 pseudo-replicates for NJ, MP and ML trees and other settings were set according to default values in MEGA v.6.0 [40]. A 50 % cut-off value was implemented for the consensus tree.

### Transcript abundance based on RNA-seq analysis

The abundances of *Hc*-*pdk-1* transcripts in different developmental stages of *H. contortus* were assessed using publicly available RNA-seq data (Haecon 5 strain, Australia; [24]). Stages evaluated were eggs, L1, L2, L3, L4 (female and male) and adults (female and male) [24]. Levels of transcription in these stages were estimated and expressed as fragments per kilobase of coding exon per million mapped reads (FPKM), as described previously [24].

### Transformation constructs

Two constructs *Hc-pdk-1*p (2975 bp)::*Hc-pdk-1* (24 bp)::*gfp*::*Ce-unc-54* t and *Ce-pdk-1*p (2958 bp)::*Ce-pdk-1* (80 bp)::*gfp*::*Ce-unc-54* t (designated pL-Hcpdk and pL-Cepdk, respectively) were made (Additional file 2). In brief, the putative promoter (2999 bp) was amplified from genomic DNA of *H. contortus* and cloned into the pPV199 vector [34] using the NovoRec PCR One-Step Directed Cloning kit (Novoprotein Scientific Inc., China), based on homologous recombination, employing primers Hc-pdk-199pro-F and Hc-pdk-gfp-R (Additional file 1). The homologous promoter region (3038 bp) was also amplified from genomic DNA of *C. elegans* and cloned into the pPV199 vector (*Bam*H1 = B and *Age*1 = A sites) employing primer pair Ce-pdk-bam-3F and Ce-pdk-bam-4R (Additional file 1).

### DNA transformation of *C. elegans*

A standard gonad microinjection method was performed in *C. elegans*, as described previously [41]. Briefly, the test constructs (pL-Hcpdk and pL-Cepdk) and plasmid pRF4 containing the marker gene, *rol-6*, were co-injected at final concentrations of 20 ng/μl and 80 ng/μl, respectively. Microinjected worms were reared on Nematode Growth Medium (NGM) plates on *Escherichia coli* OP50 lawns and maintained at 20 °C. Transformants were picked from F1 progeny, based on “right-roller” and green fluorescence protein (GFP) phenotypes, and re-plated. For the detection of spatio-temporal gene expression, transformants were anaesthetized with 10 mM levamisole, steadied on a 2 % agar pad, and assessed for GFP expression using a stereomicroscope with co-axial fluorescence, and a compound fluorescence microscope equipped with differential interference contrast (DIC) optics and a camera (Olympus BX51 Japan).

## Results

### Characterization of cDNA and phylogenetic analysis of amino acid sequence data

**Fig. 1:**
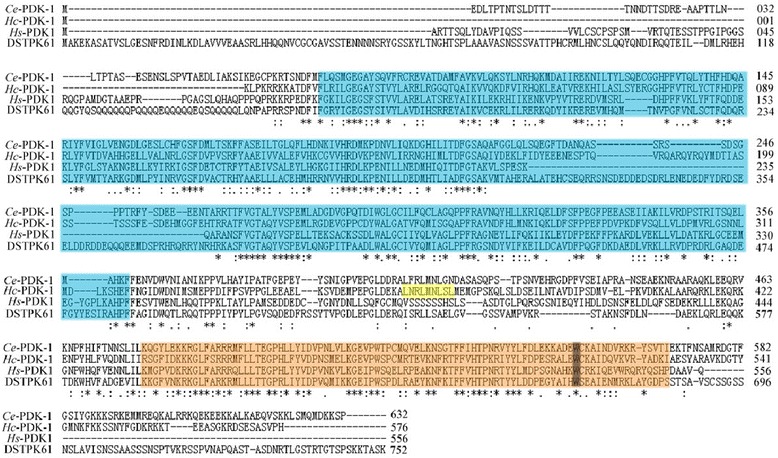
Alignment of the predicted amino acid sequence of *Haemonchus contortus* 3-phosphoinositide-dependent protein kinase (*Hc*-PDK-1) with those of *Caenorhabditis elegans* (*Ce*-PDK-1; NP_001024742), *Drosophila melanogaster* (DSTPK61; Y07908) and *Homo sapiens* (*Hs*-PDK-1; AF017995). Functional domains are boxed, with coloured backgrounds: catalytic domain (*light blue*), nuclear export sequence (*yellow*) and pleckstrin homology domain (PH; *light orange*). An invariant tryptophan (W513) is coloured in grey, which is highly conserved in the PH domain and might be involved in interaction(s) with downstream signalling proteins [42]

**Fig. 2:**
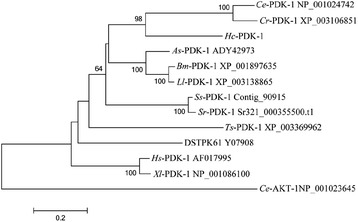
Neighbor-joining tree showing the relationship of *Haemonchus contortus* 3-phosphoinositide-dependent protein kinase (*Hc*-PDK-1) with related kinases. The tree was constructed using the Jones-Tayloe-Thornton model in the program MEGA v.6.0. Bootstrap values (>50 %) are shown above or below the branches (1000 iterations). The PDK-1 s of eight nematodes (*Caenorhabditis elegans*, *Ce*-PDK-1; *C. remani*, *Cr*-PDK-1; *Ascaris sum*, *As*-PDK-1; *Brugia malayi*, *Bm*-PDK-1; *Loa loa*, *Ll*-PDK-1; *Strongyloides stercoralis*, *Ss*-PDK-1; *S. ratti*, *Sr*-PDK-1; *Trichinella spiralis*, *Ts*-PDK-1) and three organisms other than nematodes (*Drosophila melanogaster*, DSTPK61; *Homo sapiens*, *Hs*-PDK-1; *Xenopus laevis*, *Xl*-PDK-1). *Caenorhabditis elegans Ce*-AKT-1 was used as an outgroup. GeneBank accession numbers are listed to the right of individual species names

The *Hc-pdk-1* cDNA is 1731 bp in length and encodes a protein (*Hc*-PDK-1) of 576 amino acids, which has 35–52 % similarity to homologs from *C. elegans*, *Ascaris suum*, *Loa loa*, *S. stercoralis* and *Trichinella spiralis* as well as *Homo sapiens* and *Drosophila melanogaster. Hc*-PDK-1 consists of two functional elements, namely the catalytic and pleckstrin homology (PH) domains (Fig. 1). Additionally, phospho ELM BLAST analysis (http://phospho.elm.eu.org/pELMBlastSearch.html) of *Hc*-PDK-1 predicted two phosphorylation sites (Thr226/Tyr229), inferred to play a critical role in cell signalling by phosphorylation [42]. The NetNES 1.1 Server (http://www.cbs.dtu.dk/services/NetNES/) predicted that *Hc*-PDK-1 contained a nuclear export sequence (NES) consensus, L-x(2,3)-[LIVFM]-x(2,3)-L-x-[LI], with four hydrophobic amino acid residues, which is required for the export of PDK-1 from the nucleus to the cytoplasm via nuclear transport (Fig. 1) [43]. For *Hc*-PDK-1 and other PDK-1s, an invariant amino acid (= Trp513) is shared by sequences of members of the PH domain family (see Fig. 1). The predicted *Hc*-PDK-1 protein sequence was aligned with 11 PDK-1 homologs from eight nematodes and three eukaryotes, and then subjected to phylogenetic analyses (Fig. 2). There was concordance in topology among the MP, ML and NJ trees, which showed that *Hc*-PDK-1 has a close relationship with homologs from *C. elegans* and *C. briggsae* (Fig. 2; nodal support: 98 %).

### Genomic organization, putative promoter elements and transcription

**Fig. 3:**
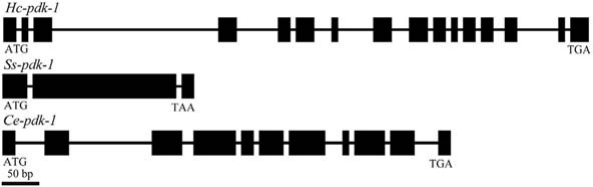
Genomic structure of *pdk-1* of *Haemonchus contortus*. Schematic diagram showing the genomic organization of *pdk-1* of *H. contortus* (*Hc-pdk-1*), *Strongyloides stercoralis* (*Ss-pdk-1*) [54] and *Caenorhabditis elegans* (*Ce-pdk-1*) [16]. Black boxes represent exons. Lines between the exons represent introns. Start (ATG) and stop (TGA/TAA) codons are indicated

**Fig. 4:**
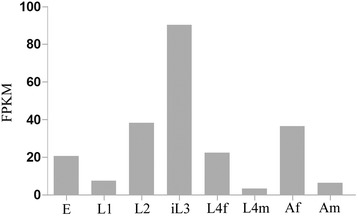
The abundance of *Hc-pdk-1* transcripts in eight developmental stages or sexes of *Haemonchus contortus*. eggs (E), first-stage larvae (L1), second-stage larvae (L2), the infective third-stage larvae (iL3), female fourth-stage larvae (L4f), male fourth-stage larvae (L4m), adult females (Af) and adult males (Am). Transcript abundance is represented as fragments per kilobase of coding exon per million mapped reads (FPKM)

We located the full-length gene of *Hc-pdk-1* in the *H. contortus* genome [24] and identified two orthologs (designated *Hc-pdk-1* and *Hc-pdk-2*). The gene *Hc-pdk-1* was 14,488 bp in length and had 16 exons (48–174 bp) that abided by the GT-AG rule [44]. However, *Hc-pdk-2* had no introns and very limited transcription, and was thus interpreted to represent a pseudogene or the result of a genomic mis-assembly. Compared with homologs from *C. elegans* and *C. briggase*, *Hc-pdk-1* had a complex exon/intron structure (Fig. 3). The 5′-genomic region upstream of the initiation codon ATG of the *Hc-pdk-1* coding sequence was 2975 bp in length and included various predicted promoter elements, such as seven E-box (CANNTG) motifs, four TATA boxes, three inverse GATA (TTATC) motifs, one CAAT (CCAAT) and five inverse CAAT (ATTGG) motifs. Study of egg, L1, L2, L3, female L4, male L4, as well as female and male adult stages of *H. contortus* (Fig. 4) revealed a high level of transcription in L3 compared with other developmental stages, indicating that *Hc-pdk-1* likely plays a core regulatory role in this stage.

### Expression pattern of the putative *Hc-pdk-1* promoter in transgenic *C. elegans*

**Fig. 5:**
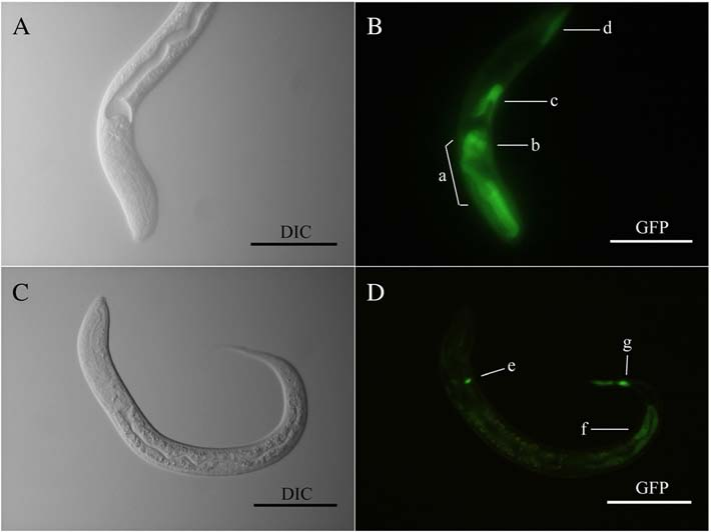
Representative expression patterns displayed in *Caenorhabditis elegans* using two GFP constructs, pL-Cepdk and pL-Hcpdk. Panels **a** and **b** Differential interference contrast (DIC) and fluorescence images of a N2 (wide type) L3 using the construct *Ce-pdk-1* p::*gfp* (pL-Cepdk), respectively. GFP reporter expression was present in head neurons (*a*), pharynx (*b*), intestine (*c*) and hypodermal cells (*d*). Panels **c** and **d** DIC and fluorescence images showing the expression of construct *Haemonchus contortus Hc-pdk-1* p::*gfp* (pL-Hcpdk) in the third larval stage (L3) of *C. elegans* (N2 strain). GFP reporter expression was present in head and tail neurons (*e* and *g*), and intestine (*f*). Scale bar = 50 μm

GFP expression driven by the *Ce-pdk-1* promoter was localized to the head and tail neurons, intestine and pharynx, consistent with a pattern reported previously [16]. Despite some minor variation among individual transgenic lines, representative gene expression driven by the *Hc-pdk-1* promoter was in head and tail neurons and the intestine (Fig. 5). Although the expression patterns for *Ce-pdk-1* and *Hc-pdk-1* were not identical, the anatomical localization of the proteins was similar (i.e. to intestine and head/tail neurons). This pattern of GFP expression was observed in all developmental stages of *C. elegans* transformed with p L-Hcpdk, including eggs, larvae and adults (data not shown).

## Discussion

The 3-phosphoinositide-dependent kinase 1 (PDK-1) is a member of the AGC family of serine and threonine kinases, and plays critical roles in physiological processes, such as metabolism, growth, proliferation and/or survival [45]. The deletion of *pdk-1* is lethal in yeast [46], *D. melanogaster* [47] and mice [48, 49], showing that this molecule is essential for the normal embryonic development. In *C. elegans*, an insulin-like signalling pathway involving PDK-1 regulates development, metabolism and longevity [16]. Loss-of-function mutation of *pdk-1* in *C. elegans* results in constitutive dauer arrest and increased lifespan [16], and the dauer stage shows similar characteristics to iL3 of parasitic nematodes [12].

Recently, homologs of genes and transcripts encoding intermediates of the insulin-like signalling pathway have been identified in parasitic nematodes. These components include the insulin-like receptor DAF-2 [29, 50], the PI3Ks protein kinase AGE-1 [30, 51] and the forkhead transcription factor DAF-16 [28, 34], suggesting that similar molecular mechanisms exist in both *C. elegans* and some parasitic nematodes. In the present study, the *Hc-pdk-1* gene was identified in genomic and transcriptomic data sets for *H. contortus*. By contrast, the *Hc-pdk-2* sequence (without introns) was found only in the genome, but not in the transcriptome, suggesting that it is a pseudogene or a misassembled genomic sequence tract.

Sequence and structural analyses showed that *Hc*-PDK-1 possesses an N-terminal catalytic domain, a nuclear export sequence and a C-terminal pleckstrin homology (PH) domain [16, 45]. PDK-1 is constitutively associated with a homo-dimeric complex through a PH domain interaction of two PDK-1 monomers; the PH domain interaction likely has significant roles in the regulation of Akt phosphorylation [52]. *Hc*-PDK-1 contains two predicted phosphorylation sites within the catalytic domain, which might be subjected to autophosphorylation [53]. Phylogenetic analyses of inferred amino acid sequence data grouped *Hc*-PDK-1 with the homologs from *C. elegans* and *C. remanei*. Additionally, PDK-1 homologs exist widely in parasitic nematodes, including *A. suum*, *L. loa* and *S. stercoralis* (confirmed by searching the National Center for Biotechnology Information databases; results not shown). These findings suggest that PDK-1 is relatively conserved and has functional similarity in various parasitic nematodes.

During key developmental transitions, including recovery of dauer larvae of *C. elegans*, and the switch from the free-living to the parasitic stage of *H. contortus*, many genes, such as *Ce-daf-7* (encoding TGF-β ligands) and the insulin-like peptide-encoding genes, are transcriptionally regulated. The current study showed that *Hc-pdk-1* transcripts are present throughout the life cycle, but have a considerably higher abundance in iL3 than other key developmental stages, which is consistent with the transcription profile of *Ss*-*pdk-1* in *Strongyloides stercoralis* [54]. This up-regulation is interpreted to relate to the switch from the free-living to the parasitic stage, in accordance with previously transcriptional evidence for *Hc-daf-2* and *Hc-age-1* [29, 30], and with a reduced metabolic rate in the L3 stage [55].

The expression of *Hc-pdk-1* predominated in the neurons and intestine of transgenic *C. elegans* larvae, which is similar to the spatio-temporal distribution of *Ce-*PDK-1 [16], where GFP was expressed under the *Ce-pdk-1* promoter in pharynx, intestine and head/tail neurons [16]. This knowledge not only contributes to understanding gene expression in time and in space, but might also assist in the prediction of protein-protein interactomes [56]. Therefore, the similar gene expression profiles of *Hc-pdk-1*- and *Ce-pdk-1*-based reporters in head/tail neurons and the intestine suggest similar regulatory functions for *Hc-pdk-1* and *Ce-pdk-1* [16]. Amphidial neurons play crucial roles in regulating the entry into and exit from dauer in *C. elegans* [57], and arrest and developmental activation of iL3 in the parasite *S. stercoralis* [58, 59]. In addition, the intestine secretes important proteins for the regulation of oocyte development [60]; this information further supports some functional similarities between *Ce-pdk-1* and *Hc-pdk-1*.

The “dauer hypothesis” proposes that the dauer larvae of the free-living nematode *C. elegans* are physiologically similar to the iL3s of parasitic nematodes [12]. Many scientists posited that this process might be regulated through similar signalling mechanisms. In parasitic nematodes, such as *A. caninum*, *A. ceylanicum* and *S. stercoralis*, LY294002, a specific inhibitor of PI3K, effectively blocks the resumption of feeding [51, 61], representing a phenotypic marker. In *S. stercoralis*, genes encoding the forkhead transcription factor *Ss*-DAF-16 have an anatomical expression pattern similar to their *C. elegans* orthologs. Furthermore, transgenes encoding *Ss-*DAF-16b with phospho-null and phospho-mimetic mutations at crucial AKT phosphorylation sites gave products with nuclear and cytoplasmic localizations, respectively [62]. Finally, mutant constructs of *Ss-daf-16b* generated a dominant-negative phenotype, including the developmental alterations of the larval intestine and pharynx, failing to arrest of transgenic larvae in the infective stage [62]. Results of studies employing *C. elegans* as a genetic surrogate indicate that central components of insulin-like signalling, such as *daf-16*, *daf-2*, *age-1* and *daf-12*, from parasitic nematodes [28–30, 32, 34, 63] have similar functional characteristics to their orthologs in *C. elegans*. This evidence supports the “dauer hypothesis” or “daf-c paradigm”, in which the activation of the L3 stage in parasitic nematodes and the recovery from dauer in free-living nematodes are governed by relatively conserved molecular mechanisms. Significantly, the CRISPR/Cas9 technology has been applied in the “model parasitic nematode” *Pristionchus pacificus* [64]. This technology might allow developmental processes of parasitic nematodes to be addressed in the near future.

## Conclusion

In the present study, we investigated a 3-phosphoinositide-dependent protein kinase-encoding gene, *Hc-pdk-1*, in the parasitic nematode *H. contortus*. We isolated and characterized the cDNA, genomic DNA and upstream (predicted) promoter elements of *Hc-pdk-1*, and assessed transcription levels of this gene in eight developmental stages/sexes. We also compared the anatomical expression patterns of *Hc-pdk-1* and *Ce-pdk-1*, and predict similar functions for these genes/gene products. Taken together, these findings provide a first glimpse of the involvement of *Hc-pdk-1* in the insulin-like signalling pathway in *H. contortus*.

## Additional files

Additional file 1:
**Primers used to isolate** ***Hc-pdk-1*** **of** ***Haemonchus contortus*** **and to make constructs for green fluorescent protein (GFP) localization in** ***Caenorhabditis elegans*** **.** (DOC 32 kb) Additional file 2:
**Cloning strategy for reporter constructs.** The constructs containing the *Caenorhabditis elegans Ce-pdk-1* and the *Haemonchus contortus Hc-pdk-1* promoters (pL-Cepdk and pL-Hcpdk) were made in the vector pPV199 [34]. Briefly, the *Ce-pdk-1* promoter region was cloned into pPV199 (*Bam*H1 and *Age*1 sites). The *Hc-pdk-1* promoter region was cloned into pPV199 by homologous recombination. (DOC 127 kb)

## Abbreviations

DIC: Differential interference contrast optics
GFP: Green fluorescence protein
JTT: Jones-Taylor-Thornton model
L3s: The third-stage larvae
ML: Maximum likelihood
MP: Maximum parsimony
NGM: Nematode growth medium
NJ: Neighbour-joining
PCR: Polymerase chain reaction
RACE: Rapid amplification of cDNA ends
UTR: Untranslated region
