# Supplementary material for: Molecular characterization of the Haemonchus contortus phosphoinositide-dependent protein kinase-1 gene (Hc-pdk-1)
Source: Parasit Vectors. 2016 Feb 3;9:65. doi: 10.1186/s13071-016-1351-6 (PMC4741024; doi:10.1186/s13071-016-1351-6)
Supplement: Additional file 1: — Primers used to isolate Hc-pdk-1 of Haemonchus contortus and to make constructs for green fluorescent protein (GFP) localization in Caenorhabditis elegans . (DOC 32 kb) [file 13071_2016_1351_MOESM1_ESM.doc]

| **Primer Name** | **Primer Sequence (5’ to 3’)** |
| --- | --- |
| Hc-pdk-F | ATGAAGCTGCCCAAACGGCG |
| Hc-pdk-R | TCAATGTGGTACAGAGGCCG |
| Hc-pdk-gw-1R | GGCCACCACGTTCGTAGCTGAGC |
| Hc-pdk-gw-2R | GTTGCCTCGAGCTTCTGATGCCG |
| Hc-pdk-pro-F | ACGGCCCGGGCTGGTGTGGAATCG |
| Hc-pdk-199pro-F | GCCTGCAGGTCGACTAGAACGGCCCGGGCTGGTGTGGAAT |
| Hc-pdk-gfp-R | GAAAAGTTCTTCTCCTTTACTCATCTTCCGCCGTTTGGGCAGCTTCAT |
| Ce-pdk-bam-3F | GGATCCGTTGCCCATGTCTTGCCAGAAAC |
| Ce-pdk-age-4R | ACCGGTGCCGCTTCACGATCCGATGTCG |
