# Supplementary figures and images for: Molecular characterization of the Haemonchus contortus phosphoinositide-dependent protein kinase-1 gene (Hc-pdk-1)
Source: Parasit Vectors. 2016 Feb 3;9:65. doi: 10.1186/s13071-016-1351-6 (PMC4741024; doi:10.1186/s13071-016-1351-6)

Additional file 2


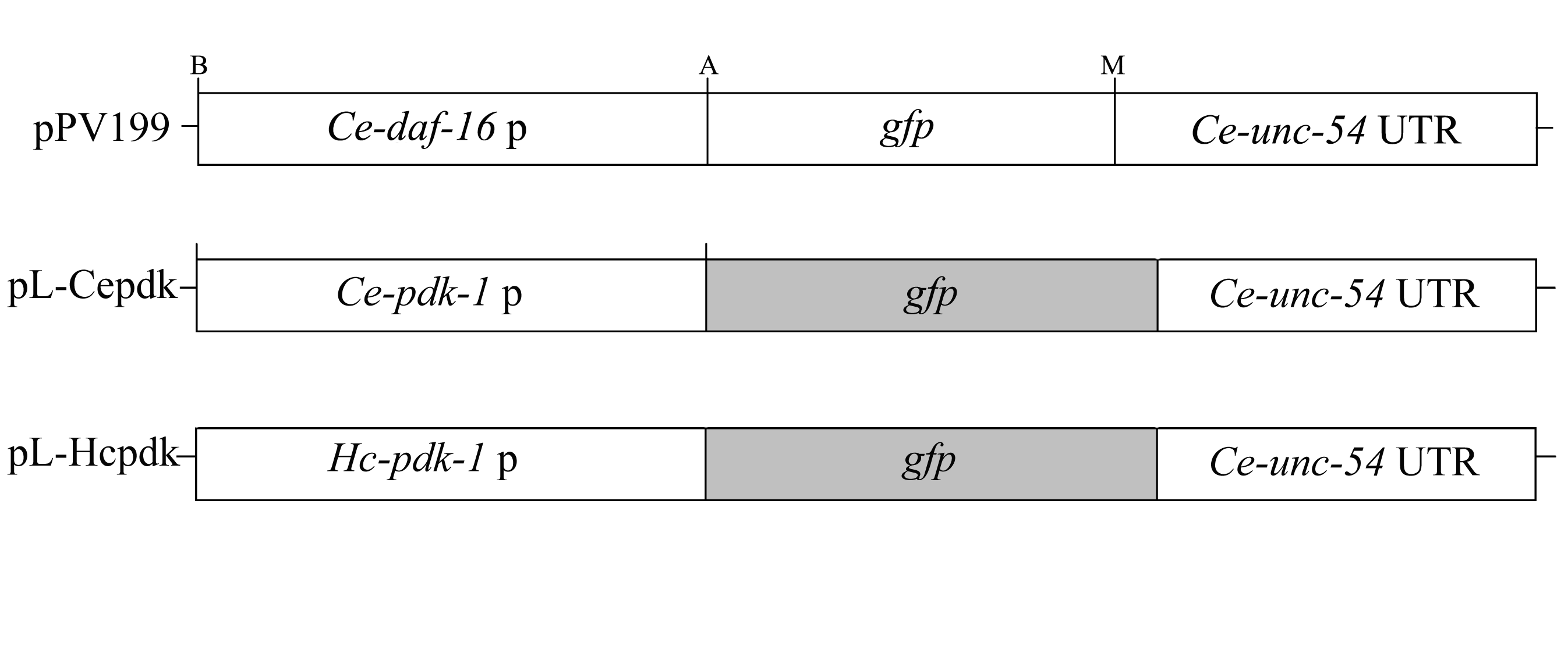

Supplement: Additional file 2: — Cloning strategy for reporter constructs. The constructs containing the Caenorhabditis elegans Ce-pdk-1 and the Haemonchus contortus Hc-pdk-1 promoters (pL-Cepdk and pL-Hcpdk) were made in the vector pPV199 [34]. Briefly, the Ce-pdk-1 promoter region was cloned into pPV199 (BamH1 and Age1 sites). The Hc-pdk-1 promoter region was cloned into pPV199 by homologous recombination. (DOC 127 kb) [file 13071_2016_1351_MOESM2_ESM.doc]
